# Alibert

**Published:** 1838-01-31

**Authors:** 


### MEDICAL EXAMINER.

PHILADELPHIA.

Wednesday, January 31, 1838.

### ALIBERT.

We noticed hastily in our last number the death of this eminent physician, who expired at Paris in November last, at the age of seventy. We shall now gratify the curiosity of our readers by a cursory sketch of his life. He was born in the village of Ville-franche, in what was formerly Haute Guyenne, in France. He was early destined for the Church, but the troubles of the revolution breaking out about that time, directed his attention in another channel. He removed to Paris, and became a pupil in the *Normal* school, an institution which, during its brief existence, gave considerable impulse to national education. It was not until this school was closed that Alibert devoted his exclusive attention to medicine. The peculiar qualities of his mind, and the singular benevolence of his disposition, peculiarly adapted him for the exercise of the active duties of his profession. Many of his productions, and particularly those by which he is best known, were begun and completed during the first few years of his practice. We may mention among others, his Treatise on Malignant Fever, which has been long regarded as a standard work; his Essay on Therapeutics, based on the physiology of Bichat; a system of nosology which first displayed that peculiar talent for classification, afterwards so conspicuous in his elaborate production on the Diseases of the Skin; to these we may add numerous memoirs, eulogies, and other papers prepared for the Medical Society of Emulation, of which he was one of the most distinguished and active members. The first sketch of his great work on Cutaneous Affections appeared in 1806. It was not until 1832, that it was finally completed. The skill, fidelity and effect with which the illustrations of this noble work are executed are well known. This was done by Alibert at his own risk, and at an expense it is said of 300,000 francs. Willan had already acquired a reputation in this department, and as works of art doubtless his plates possess much merit; but the superiority of vivid and natural delineation appears by general consent to be allotted to Alibert. The descriptions of the latter are also rendered more graphic and highly interesting by the vivacity of his style, and the richness of his imagery. The prominent fault of his nomenclature is excess of refinement, and of ingenuity, which unfortunately is better calculated to fatigue than to assist the memory of the student.

His Treatise on the Passions, a work of a more purely literary character, was written at a later period. It is but justice to Alibert to confess that in treating of the Passions he has adopted a higher moral tone than is characteristic of the French School in general, and that his views appear to be much influenced by his early religious impressions.

For the last twenty years of his life, Alibert occupied the post of physician in chief to the Hospital of St. Louis. This was the great scene of his labours and of his glory. Here he established his clinic on Cutaneous Diseases, and here in the open air, like one of the ancient academic sages, drawing his pupils around him, he exhibited and explained to them the cases, the number of which sufficed to illustrate the most exact classification, and by the power of his eloquence recreated this, his favourite branch of scientific investigation.

In his private character Alibert was distinguished for benevolence and kindness. His especial delight was giving aid and countenance to the junior members of the profession, and he regarded his pupils as his children. In his professional relations he was liberal and disinterested; all who sought his aid, whether rich or poor, were received with courtesy and kindness; and the importance of the malady, not the rank of his patient, was the rule and measure of the attention which he paid the case. Some of his more direct acts of benevolence were managed with extreme delicacy. If he discovered any artist, or literary man, who was reduced in his circumstances, he sent by some unknown hand, and under a fictitious name, considerable sums of money. Where the transaction was open, and the pride of the recipient made him insist on giving his note, it was either destroyed or cancelled. More than one hundred obligations thus mutilated were found among his papers.

*Eloges* were delivered over his grave by M. M. Pariset, and Cruveilhier.

---

We regret that we are again obliged to disappoint our correspondents. The lectures and reports of the Hospitals are so numerous, and are of such paramount importance, that we are compelled to exclude for the present, much excellent and acceptable matter.

